# Telestration with augmented reality in minimally invasive and robotic-assisted surgery: a scoping review

**DOI:** 10.1007/s00464-025-12380-2

**Published:** 2025-11-18

**Authors:** Amila Cizmic, Frida Häberle, Anas A. Preukschas, Laetitia Hampe, Vasile Bintintan, Thilo Hackert, Manish Chand, Felix Nickel

**Affiliations:** 1 Department of General, Visceral and Thoracic Surgery, University Medical Center Hamburg-Eppendorf, Hamburg, Germany; 2 Department of General, Visceral, and Transplantation Surgery, University Hospital Heidelberg, Heidelberg, Germany; 3 Department of Surgery, University Hospital Cluj Napoca, Cluj-Napoca Napoca, Romania; 4 Division of Surgery and Interventional Science, University College London, London, UK

**Keywords:** Telestration, Augmented reality, Minimally invasive surgery, Robotic-assisted surgery, Surgical training, Medical education

## Abstract

**Background:**

Minimally Invasive Surgery (MIS) and Robotic-Assisted Surgery (RAS) offer advantages over open surgery, including smaller incisions and quicker recovery. However, the learning curve in MIS and RAS presents several challenges, primarily due to their reliance on verbal instructions. MIS and RAS training is continually evolving, with new educational and simulation models emerging in recent years. Telestration, combined with augmented reality (AR), enables annotating videos and images as active feedback during surgery. These modalities have been used in the operating room to highlight anatomical structures, aiding visual communication and reinforcing verbal communication in MIS and RAS. This study aimed to provide a scoping review of the use, current applications, and development of telestration and with AR in MIS and RAS.

**Methods:**

A scoping review was conducted using the Joanna Briggs Institute methodology and the PRISMA extension for scoping reviews (PRISMA-ScR) 2018 statement and recommendations. Two researchers independently searched the literature in the following databases: PubMed, the Association for Computing Machinery (ACM), the Institute of Electrical and Electronics Engineers (IEEE), and Google Scholar.

**Results:**

From a total of 625 screened studies, 33 were included in the scoping review based on relevance, reported innovation, clinical and surgical implementation, and potential benefits in MIS and RAS. Intraoperative surgical guidance using telestration with AR has already been implemented in the MIS and RAS fields, such as urology, colorectal surgery, gynecology, and orthopedics. Telestration with AR is increasingly used in MIS and RAS simulation and training. This technology optimizes communication during surgical procedures, reduces complication rates, and enhances performance, potentially leading to improved patient outcomes.

**Conclusion:**

Telestration with AR is an innovative tool in MIS and RAS that improves surgical training, communication, and idea exchange, sometimes replacing on-site mentoring.

**Graphical Abstract:**

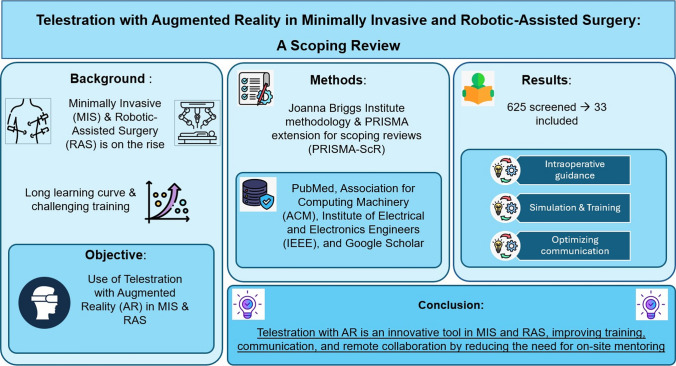

**Supplementary Information:**

The online version contains supplementary material available at 10.1007/s00464-025-12380-2.

## Identifying the Scoping Review Question

Minimally invasive surgery (MIS) has been favored over open surgical procedures due to its numerous benefits, including smaller incisions, reduced postoperative pain, a shorter hospital stay, and a faster recovery to normal conditions [1]. However, the learning curve for MIS is lengthened by the lack of three-dimensional (3D) vision, a narrow two-dimensional (2D) field of view, pivoting and fulcrum effects, reduced haptic feedback, and limited degrees of freedom of movement [2]. Surgical teaching and proctoring in MIS are further hindered because intraoperative instructions can be only given verbally, not visually. For example, pointing at structures on the operative screen is impossible due to distance, ergonomic constraints, and sterility concerns. This generates the need for innovative and accessible teaching tools. The shift from open to MIS requires practical and accessible teaching methods [3].

Practical intraoperative training and guidance in surgery are based on the quality of communication between mentors and trainees and have traditionally been conducted on-site. Communication errors can lead to prolonged operating times, increased stress in the operating room (OR), and adverse events. This can be particularly problematic in MIS, where trainees must rely solely on verbal communication, as mentors have limited means of directly interacting with the surgical field when trainees use surgical instruments. Furthermore, the COVID-19 pandemic negatively affected surgical training by limiting travel possibilities and reducing access to on-site mentoring and proctoring by expert surgeons [4]. Additionally, the travel restrictions could make it challenging to provide urgent, complex, and high-quality surgical services in remote and rural areas with limited resources in various surgical specialties [3]. This adds to the global shortage of surgical expertise. New technological innovations in surgical telementoring can help address these challenges [5].

Although on-site mentoring is considered the standard for surgical training, no difference in knowledge or skill acquisition has been reported between telementoring and on-site mentoring [6]. Surgical telementoring involves using information technology to provide real-time guidance and technical assistance from an expert surgeon in a different geographical location during the performance of surgical procedures [7]. Telementoring has demonstrated its reliability, efficiency, and cost-effectiveness as an educational tool and mentorship model [7].

Robotic-assisted surgery (RAS) is an evolution of MIS and offers many advantages compared to open surgery [8, 9]. However, one major problems is the limited number of well-trained robotic surgeons [10]. RAS requires different skills and training compared to open and laparoscopic surgery. Therefore, a specific program for RAS training is necessary to develop competence and guarantee patient safety [11]. Nowadays, telementoring in RAS is a viable alternative to traditional on-site mentoring, allowing more efficient communication between physicians [12].

Therefore, this scoping review was guided by the following research questions:

1. What are the current applications of telestration with augmented reality (AR) in MIS and RAS?
2. What are the reported benefits, challenges, and limitations associated with telestration with AR in MIS and RAS?

### Definitions

**Fig. 1:**
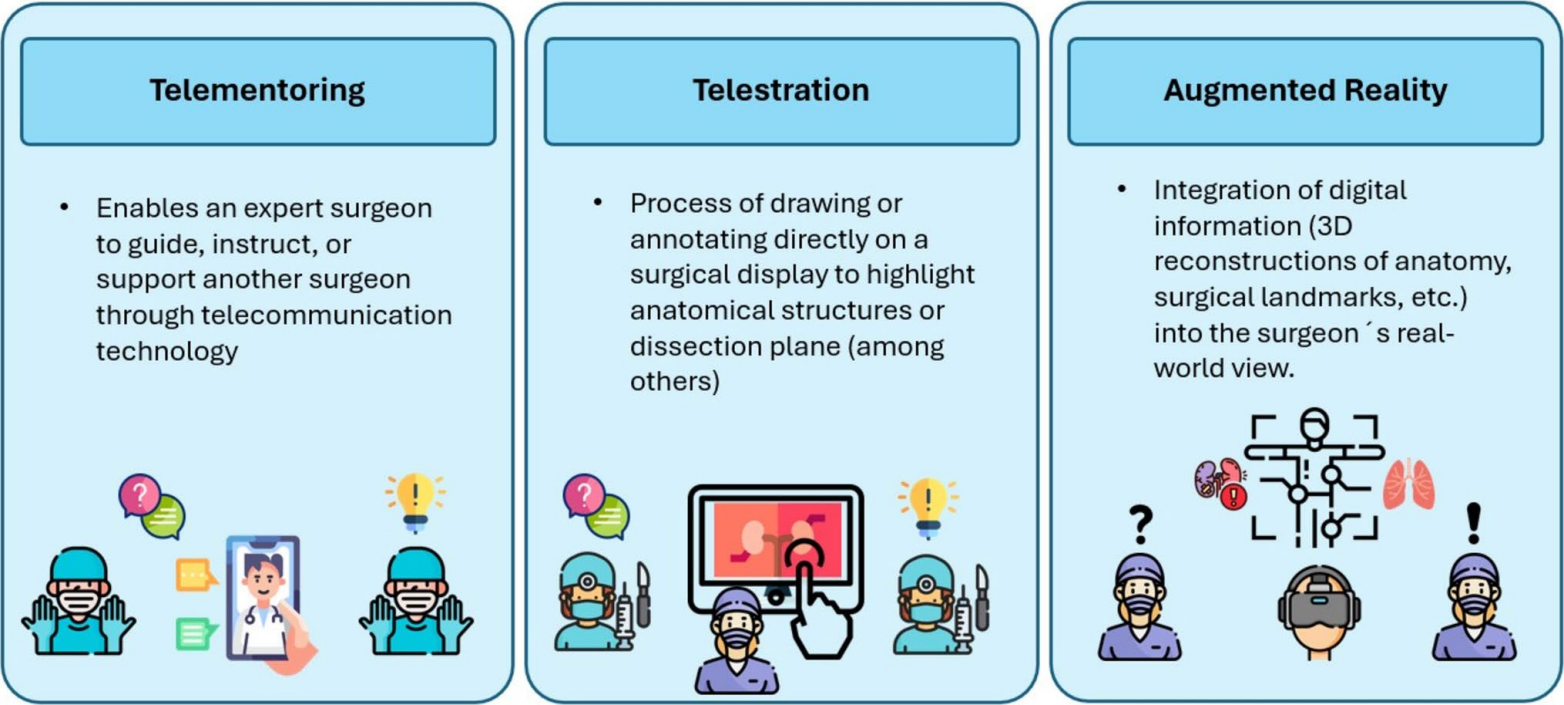
Visual presentation of the telementoring, telestration and augmented reality definitions

To improve the readability of the manuscript, it is essential to define the three most commonly used terms: telementoring, telestration, and AR. Telementoring is a concept within telemedicine where an expert surgeon guides another at a different geographical location (remotely) [13]. Telestration is a technique for enabling the drawing of freehand markups, also known as annotations, by displaying hands or other structures over images or videos in real time [14]. AR is a hybrid system that integrates virtual elements and reality, enabling interactions between the mentor, the mentee, and the surgical field at all times [15]. The three crucial definitions of the manuscript are presented in Fig. 1.

### The role of telestration with augmented reality in MIS and RAS

While telementoring is restricted to remote proctoring, telestration with AR can be used on-site and remotely. Telestration is considered an integral function of telementoring surgical systems [6]. Telestration, combined with AR in MIS and RAS, is becoming a more practical and cost-effective tool for teaching advanced surgical skills worldwide and for delivering surgical care to underserved areas [3, 16, 17]. Time, potential cost reductions, and more efficient surgical education are only a few of the many evident advantages of this evolving technology [18].

Telestration offers remote and on-site mentoring and instruction through enhanced communication, including verbal and visual cues via images or video conferencing. It can be projected in 2D and 3D models with AR [19]. Technological advances have progressed even further, allowing for the live streaming of the surgeon’s hands on the operative screen, enabling direct, live instructions and intraoperative guidance [20]. AR can register both virtual and real items in 3D, providing interactions at all times [15]. Whereas telestration with AR can be used globally in remote teaching settings, it can also be used for on-site training where the mentor and the trainee are both in the same OR. The mentor can assist, advise, and direct the trainee’s next step during the surgery with visual guidance, using a virtual hand or other annotations on the operative screen and verbal instructions, thereby avoiding unnecessary instrument movements, prolonged operative times, stress, and potential complications.

### The first uses of telestration in MIS/RAS

One of the first applications of telestration in MIS/RAS was in 2001 with the Socrates system (Computer Motion Inc.) in combination with the Zeus system. It was the first telecommunication tool that allowed telementoring and telemanipulation over transatlantic distances. It provided a two-way video and audio communication between a mentor and the mentee on-site. The telestration tool used an electronic stylus to pinpoint anatomical structures and control the endoscopic camera. The first procedure under this telementoring setup was a laparoscopic cholecystectomy performed in October 2001 in Strasbourg, France, under the mentoring of a professor of surgery located in New York, USA [21].

### Objectives

This scoping review aimed to identify and synthesize the latest available evidence on telestration with AR in the fields of MIS and RAS. Additionally, the aim was to recognize their current application in the OR and their reported telementoring possibilities.

## Materials and methods

The scoping review has been conducted according to the recommended Framework for Scoping Reviews [22, 23] and the Joanna Briggs Institute (JBI) methodology [24]. The framework used for the reported scoping review is presented in Appendix 1. The presented scoping review was conducted according to the Preferred Reporting Items for Systematic Reviews and Meta-Analyses extension for Scoping Reviews (PRISMA-ScR) statement [25]. Appendix 2 provides the PRISMA-ScR checklist in detail. The scoping review team included junior and senior general and visceral surgeons with clinical and practical knowledge of MIS and RAS, as well as the implementation of telestration with AR in MIS and RAS. Two reviewers (AC and FH) independently conducted descriptive and full-text analyses of the characteristics of the included publications.

### Search strategy

To identify and synthesize the latest scientific evidence and current applications of telestration with AR in MIS and RAS, a comprehensive literature search was conducted across the following databases: PubMed, Association for Computing Machinery (ACM), Institute of Electrical and Electronics Engineers (IEEE), and Google Scholar. The literature search included studies published from June 2005 to March 2025. The study was conducted using a predefined set of keywords: "telestration", "augmented reality", "telementoring", "minimally invasive surgery", "laparoscopy", "robotic-assisted surgery", and "surgical training". The entire search strategy across all databases is presented in Appendix 3.

### Charting data

For data extraction, we conducted a literature search using the following information for each publication: title, year of publication, country, type of telestration tool, participants, intervention, distance (for tools with telementoring function), factors/outcomes, and price of the telestration tool, when reported.

### Inclusion and exclusion criteria

Studies were eligible for inclusion if they mentioned using and implementing telestration with AR in MIS or RAS, regardless of the surgical field. Studies that discussed the advantages, limitations, and potential applications of video annotation tools with AR in MIS or RAS, as well as telementoring systems, were also included.

Studies were excluded if they were written in a language other than English. Systematic reviews, scoping reviews, comments, opinion papers, editorials, publications of abstracts alone, and book chapters were also excluded from the study. Articles were excluded if they did not include or mention telestration tools with AR in their materials and methods. Studies that were not relevant to our topic were excluded.

### Screening process

The studies were selected based on their reported innovation, implementation of telestration systems with AR, and clinical and educational benefits. The aim was to systematically assess and present the diverse spectrum of studies demonstrating telestration and AR in surgical proctoring and remote surgical guidance.

## Results

**Fig. 2:**
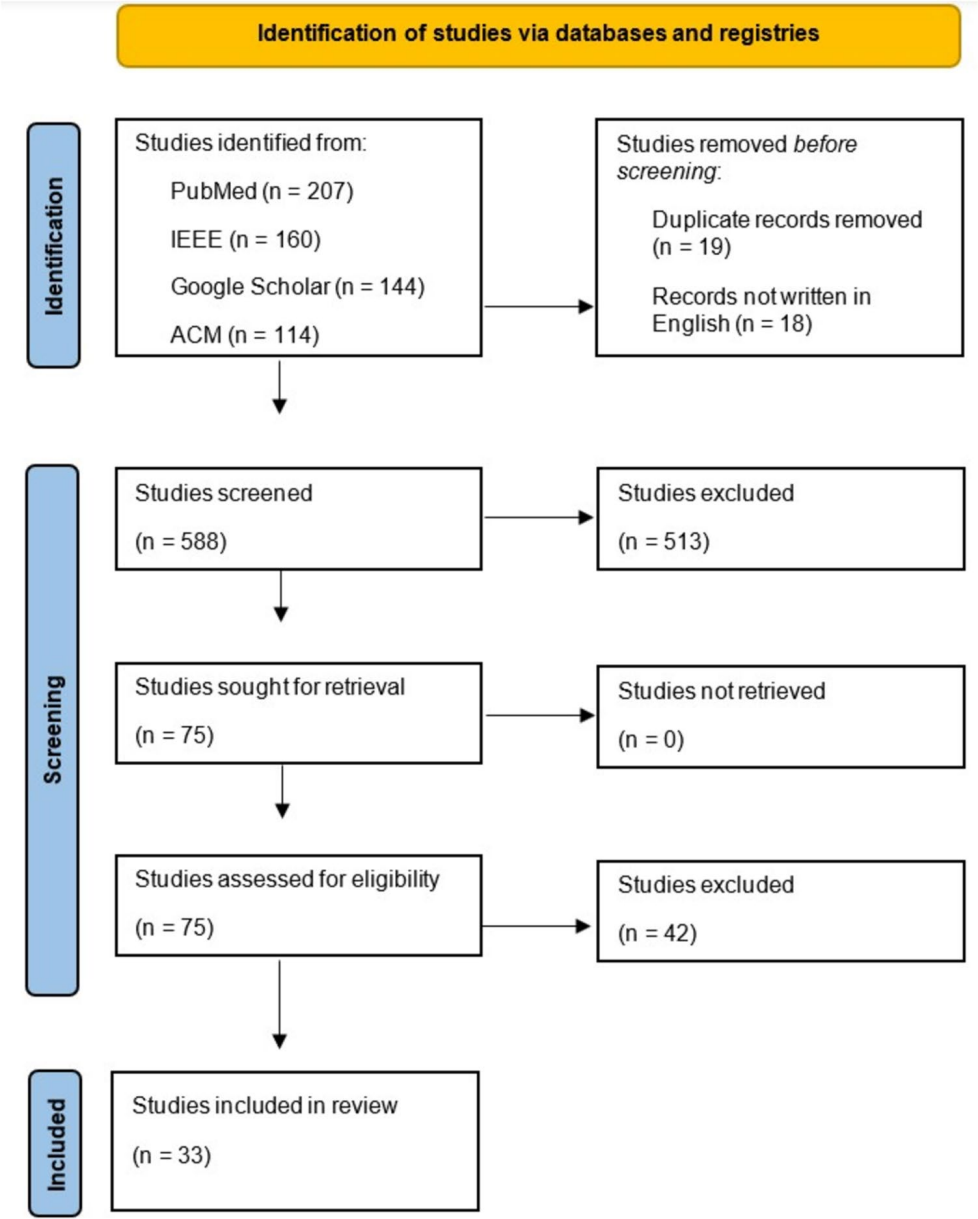
PRISMA-ScR Preferred Reporting Items for Systematic Reviews and Meta-Analyses for Scoping Reviews flow diagram of the search and study selection process [67]

A total of 625 studies were initially identified for inclusion in the analysis. The abstracts and titles were screened for consideration for the scoping review (Fig. 2). Thirty-seven studies were excluded due to language requirements and duplicate records. After screening, we excluded 513 articles for lack of relevance to our topic or publication type, and 42 papers were excluded after eligibility assessment. The remaining 75 full texts and their references were reviewed to determine if other studies should be included in the scoping review. Finally, we included the 33 most informative studies focusing on the application of telestration with AR in MIS and RAS to provide a comprehensive overview of this topic [19, 20, 26–56].

### Characteristics of the included studies

All 33 studies included in this scoping review were original research articles. The telestration tools with AR were used in surgical simulation (animal models and synthetic simulators) and the real OR with actual patients. The authors typically discuss the experience, advantages, and limitations of telestration tools in AR. Appendix 4 provides a detailed presentation of all included studies.

### Categorization of the included studies

**Fig. 3:**
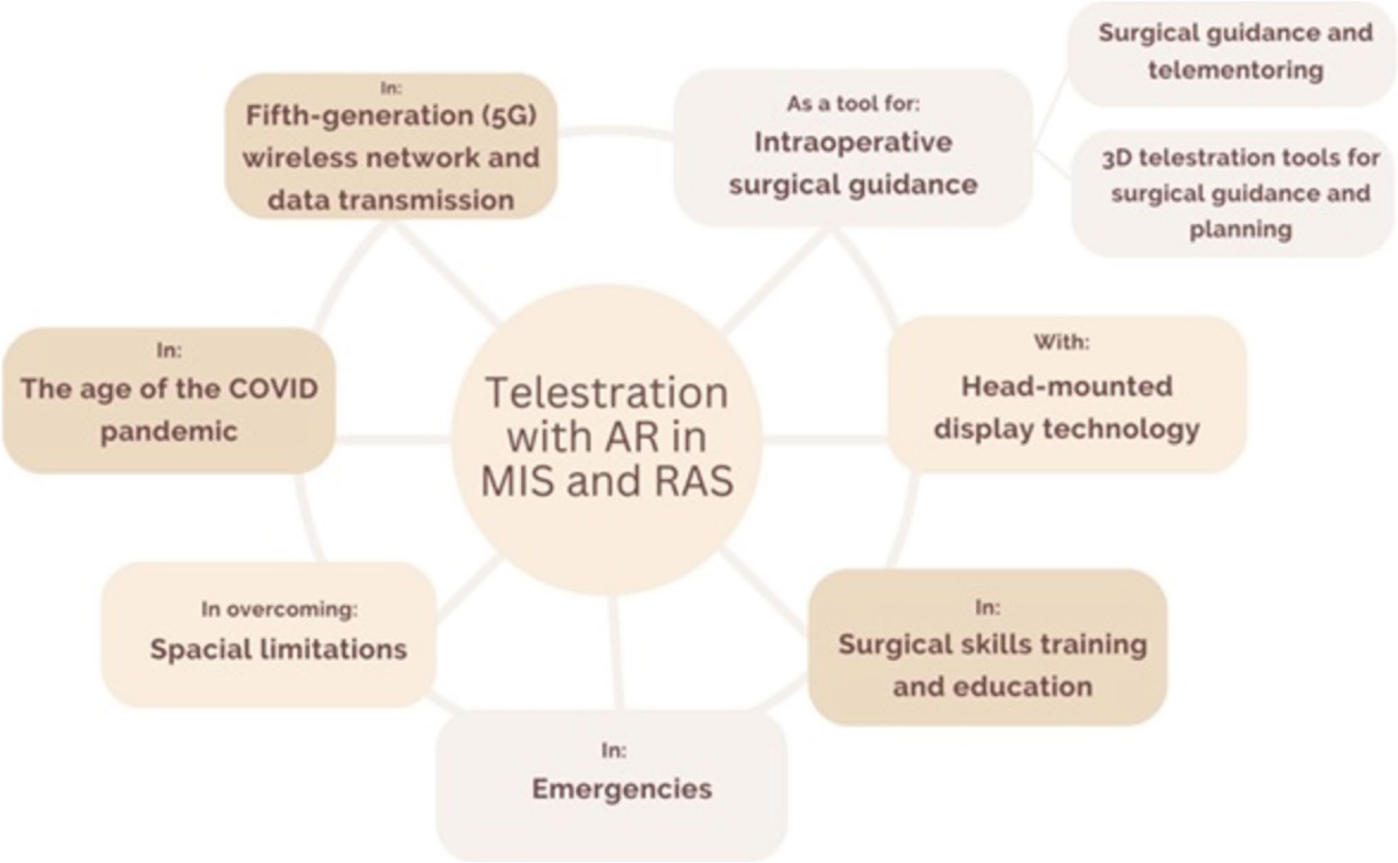
Categorization by main topics of the included studies

To categorize the primary applications of telestration with AR in MIS and RAS, as mentioned in the included studies, we organized them into the following categories, as presented in Fig. 3. To provide a clear overview of the different categories of the included studies and the various application forms of telestration with AR in MIS and RAS, the following structure of reporting has been chosen: a) Telestration with AR as a Tool for Intraoperative Surgical Guidance (with subcategories: Surgical Guidance and Telementoring and 3D Telestration Tools for Surgical Guidance and Planning), b) Telestration with AR and Head-Mounted Display Technology, c) Telestration with AR in Surgical Skills Training and Education, d) Implementation of Telestration with AR in Emergencies, e) Telestration with AR in Overcoming Spatial Limitations in MIS and RAS, f) Overcoming Challenges of Surgical Training in the Age of the COVID-19 Pandemic, and g) Fifth Generation (5G) Wireless Network and Data Transmission.

#### Telestration with AR as a tool for intraoperative surgical guidance

Telestration, combined with AR, enables highlighting critical anatomical structures or pathologies within the field of view during surgery. Therefore, it can potentially be helpful for intraoperative guidance [57].

In 2004, Niemeyer et al. introduced one of the first descriptions of devices using telestration with AR in MIS and RAS. The authors developed the Two-Handed Universal Master Project (THUMP), which consists of a haptic console paired with stereoscopic goggles for RAS training and simulation. This console offered an opportunity for advanced mentoring, where a mentor could share control with a trainee and provide insightful feedback via the telestration tool with AR [58].

Since then, the potential of telestration with AR to merge virtual and real objects has been a research target, especially in surgical simulation training, where students and surgeons must prepare themselves for real operations. Such a safe training environment is crucial in overcoming critical points of the learning curve in MIS and RAS [2].

### Surgical guidance and telementoring

In recent years, several studies have begun to address the subject of telestration and AR as intraoperative surgical guidance [26, 29, 32, 33]. One study presented the use of a second-generation telementoring interface developed by Intuitive Surgical Inc., called Connect [33]. Connect is a software program that adds remote mentoring capability to the da Vinci Si Robot, allowing surgical mentoring from outside the OR. It utilizes Internet connectivity to incorporate three primary features: one-way video, two-way audio, and telestration. In 2014, Shin et al. compared remote mentoring with Connect in RAS procedures in urology with in-room mentoring. The parameters, including operating time, blood loss, and robotic skill assessment, were evaluated. Remote mentoring with the Connect telestration interface tool showed comparable results to in-room mentoring, providing a promising solution for remote assistance and telementoring in RAS.

Surgical guidance via telementoring was also used in pediatric surgery. They performed video-assisted thoracic surgery, lower lobectomy, temporary and permanent gastric stimulator placements, and two laparoscopic inguinal hernia repairs. They compared two different telementoring technologies (system connected to Skype™ and VisitOR1®). All procedures were completed successfully without complications and loss of transmission [36].

Similarly, Hinata et al. compared two surgeons performing 30 robotic-assisted radical prostatectomies under the guidance of an audiovisual telementoring system with telestration and AR to two surgeons performing the same surgery with in-room mentoring [29]. Compared to the direct mentoring group, remote assistance with a web-based audiovisual telementoring system showed no significant differences in several parameters reflecting surgical outcomes, including operating time, complication rate, early continence status, and positive margin rate.

Another study, published by Shafa et al., found that remote video-conferencing for video-based coaching and telestration improved the operative performance of early career surgeons [52]. Remote coaching was implemented using the Zoom platform, allowing for freehand drawing annotations on the surgical field. Most participants found this tool useful and were satisfied with the coaching program offered on the virtual platform. The study has shown that even platforms familiar to most people, such as Zoom, can have a positive impact on the remote coaching of surgical procedures.

Finally, Takemasa et al. conducted a study on teleproctoring for MIS across Japan to assess its clinical feasibility using an ultra-low-latency communication system and shared internet access (SIA) [53]. Seven hospitals participated and tele-proctored with Sapporo Medical University. The latency of telestration between the two locations was measured by detecting the use of an annotation pen; annotations were made and transmitted in real time to the surgeon’s monitor. They concluded that this system offers a sustainable approach to surgical education, particularly in rural areas and in countries with limited medical infrastructures, helping reduce healthcare disparities.

### 3D telestration tools for surgical guidance and planning

In 2008, Ali et al. introduced one of the first 3D telestration prototypes for RAS, developing a video algorithm that translated a 2D telestration provided by the mentor station into a 3D telestration for trainees at the robotic console in the da Vinci visual field [27]. The preliminary results were collected using inanimate models, and the prototype device proved usable, reliable, and accurate.

Another study presented 3D semitransparent proctoring ghost tools overlaid on the surgeon’s field of view. The three types of 3D ghost tools (3D pointers, 3D cartoon hands, and 3D instruments) were first examined and compared to conventional 2D telestration tools in four dry-lab exercises [32]. The dry-lab exercises targeted technical skills related to using the da Vinci Surgical System. The 3D ghost tools were used to support proctoring. The 26 proctor-trainee pairs who participated in the study assessed the utility and efficiency of the new proctoring tools.

A year later, 3D proctoring tools were assessed on realistic surgical tasks, such as tissue dissection and suturing, in a live porcine model using the da Vinci Xi Surgical System [19]. Jarc et al. employed telestration with AR techniques in mentor–trainee training scenarios for RAS, where the mentor could control the 3D ghost tools rendered as augmentations on the trainee’s immersive display. The preliminary evaluation, conducted with a limited number of participants, found that both the mentor and trainee favored the AR-based mentoring technique [19]. By visualizing additional information on the target anatomy or lesion via AR, ideally registered with the laparoscopic video, AR-based surgery guidance can improve the surgeon’s situational awareness and ease intraoperative decision-making.

Surgical planning tools have also been developed together with smartphones and AR technology. Yang et al. presented a mobile AR-based cardiovascular surgical planning tool. CardiacAR is an iOS application that enables an AR view of a patient’s 3D heart models in real-life environments. It allows users to annotate and perform virtual omnidirectional slicing of anatomical structures [44].

In the field of neuroendoscopic surgery, Tanaka et al. published a study in 2024, examining a telestration system designed to enhance communication between trainees and mentors [56]. Annotations were made directly on the surgical field for endoscopic transsphenoidal surgery (ETS). The systems used in the study were NUCLeUS and ADMENIC ANNOTATOR. The mentor was able to draw annotations on a tablet, and the image was displayed on a second monitor. The surgery was performed while viewing the primary monitor and the transmission monitor. The study concluded that using this system improves communication and contributes to safer, more precise surgeries, thereby reducing operating time.

#### Telestration with AR and head-mounted display technology

In 2023, a study presented a telestration with an AR tool called AR Head-Mounted Display (ARHMD) for surgical teleconsulting and real-time telestration [47]. Twelve attending surgeons participated in the study, simulating six clinical scenarios: three for breast surgery and three for abdominal and pelvic surgery. The addressed situation involved a surgeon consulting a radiologist when difficulties arose in finding non-visible structures during surgery that had been previously identified on a preoperative image. A video of the procedure is captured in real-time with a head-mounted camera and shared directly with a colleague in a different room. The participants could communicate through deictic referencing employing physical fingers or a virtual pointer. The use of virtual arrows and annotations was also possible.

Another HMD telestration tool is the HoloPointer, which provides intraoperative surgical guidance and AR annotation on laparoscopic monitors [51]. The participants used mixed reality glasses (HoloLens) to see VR pointers directly on the laparoscopic screen. This system operates via verbal commands and head movements, ensuring a sterile workflow in the OR. The study evaluated the surgical influence of HoloPointer in 32 elective laparoscopic cholecystectomies (LCs). The subjective surgical performance improved by 84.6% among participants using this AR telestration system.

The use of this technology has also been reported for teaching basic surgical skills. In 2022, Neves et al. compared the suture performance of two groups of medical students. One group received traditional mentoring, while the other received mentoring via telestration on a head-mounted device with AR capabilities. A 2D mouse was used for visual instructions, such as pointing and adding text and objects to the participant’s screens. The students in the telestration group completed the exercises faster, but there were no differences in the quality of the surgical gesture or the tension of the suture [42].

In 2024, Liu et al. explored the application of AR for teaching the anatomy of spinal tumors and a percutaneous vertebroplasty [54]. A head-mounted device was employed to create different learning scenarios. The program features a visual projection that allows users to see structures and receive guidance from tutors during AR procedures. The model offers interactive functions, including finger dragging, zooming, and tapping. For the surgical simulation, they reconstructed instruments used in vertebroplasty, including drapes, anesthesia syringes, needles, puncture cannula, and more. The study is the first reported attempt to apply AR technology in medical education for spinal tumors. It concluded that this method offers greater teaching effectiveness than traditional approaches, making medical education more accurate, efficient, and intuitive.

#### Telestration with AR in surgical skills training and education

Surgical training plays an essential role in developing and refining technical skills through the guidance of experienced mentors, and nowadays, with the help of telestration and AR. Regardless of the complexity of the procedures, from basic surgical skills to more advanced procedures, the use of telestration and AR helps trainees and mentors communicate efficiently, provides visual aids, and engages in hands-on practice [49, 59].

Telestration with AR can compensate for the otherwise limited communication possibilities in MIS and RAS training by enabling a visual communication dimension, rather than relying solely on verbal interaction. This can be achieved both on-site and remotely, overcoming the logistical obstacles of traditional mentoring and proctoring. Several self-developed and commercially available systems can now overlay virtual hand movements and gestures or paint, draw resection lines, and mark risk structures as augmented reality overlays in real-time during MIS and RAS [28, 30].

**Fig. 4:**
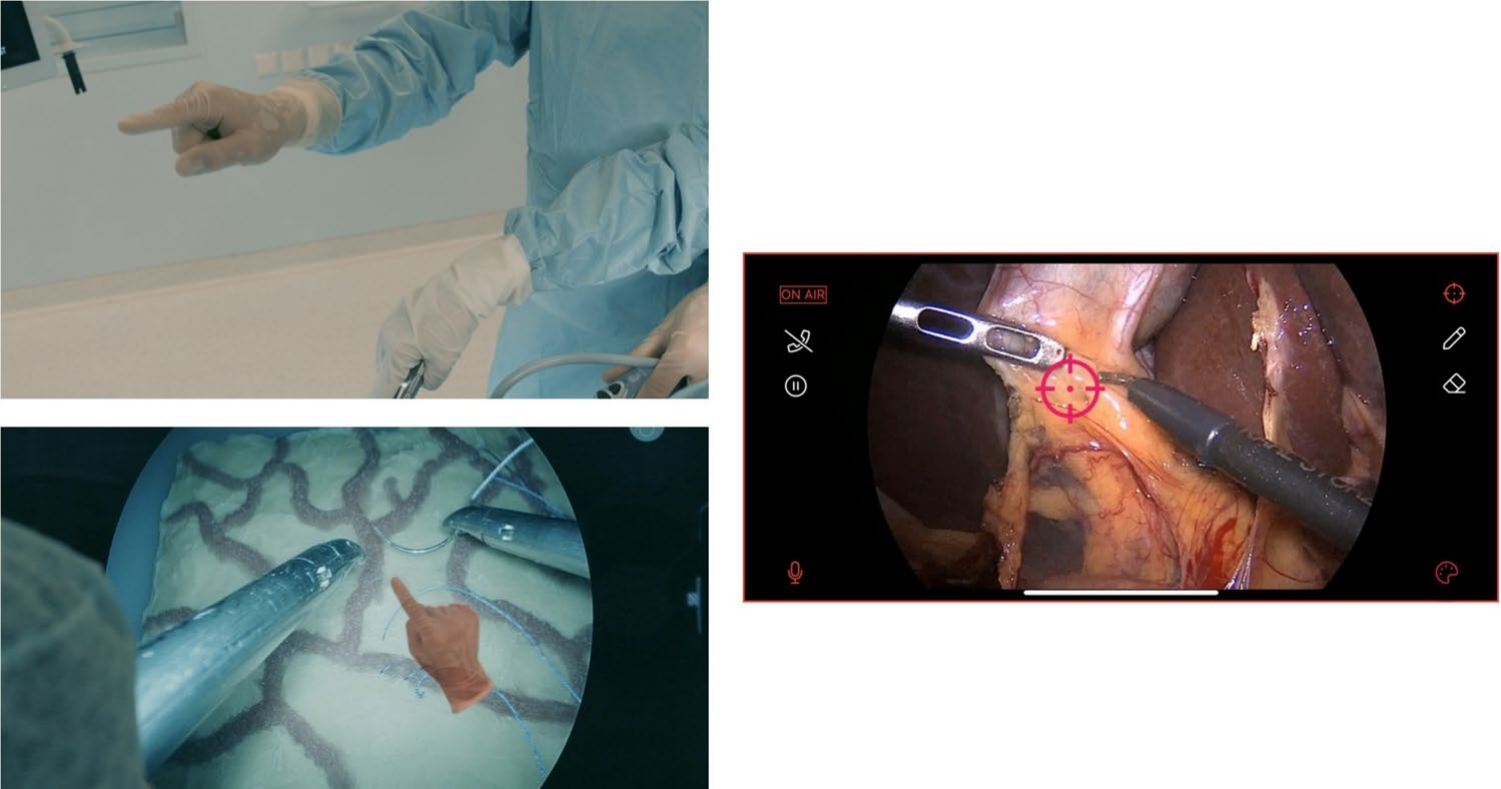
Practical use of iSurgeon in simulation training (on the left) and on a laparoscopic cholecystectomy (on the right)

The iSurgeon system provides real-time telestration with an AR overlay of the mentor’s hands on the operative screen in MIS (Fig. 4) [20, 45]. The study was conducted on participants with varying laparoscopic experience who performed four laparoscopic stitches and knots using the C-loop technique. During the training, a tracking motion system consisting of an NDI Polaris and a Microsoft Kinect v1 IR camera was used to track joint angles and upper extremity motion. The iSurgeon could discriminate between different experience levels, therefore establishing construct validity. The OSATS score, the current gold standard for assessing technical skills in laparoscopic suturing and knot tying, is highly correlated with the iSurgeon parameters. The iSurgeon could generate automated real-time feedback based on expert models. This might have a positive impact on the trainees, specifically by shortening their learning curves for laparoscopic tasks.

The iSurgeon was used and assessed in a randomized controlled study with a crossover design [43]. Sixty medical students were randomized into two groups. The first group received only verbal guidance, while the second group received additional telestration with AR (iSurgeon) during an LC. The iSurgeon group showed significantly better GOALS and OSATS scores, as well as a lower complication rate, compared with the group receiving only verbal guidance.

The most recent studies regarding the iSurgeon system were published in 2023. Felinska et al. performed a randomized controlled crossover study to analyze the effects of telestration-guided instructions on gaze behavior during MIS training [48]. The gaze behavior was captured using pupil-core eye-tracking glasses. The participants were forty laparoscopically naive medical students randomized into two groups (AR telestration and verbal instructions). The trainees completed different basic laparoscopic tasks and one LC at the end of their training. Parameters such as gaze latency, gaze convergence, and collaborative gaze convergence were significantly lower in the iSurgeon group compared to the verbal instruction group. The telestration group also obtained significantly higher LC scores, indicating that telestration with AR successfully improved surgical performance and confirming that this tool is effective when using gaze guidance in MIS.

The latest study on the use of iSurgeon as an AR telestration tool is a randomized controlled study that included forty medical students [50]. Half of them received both verbal and visual mentoring with the iSurgeon tool, while the rest received verbal instructions only. Both groups performed a baseline LC and repeated the procedure ten times. Trainees who received mentoring with telestration achieved the Critical View of Safety (CVS) more frequently, showed better MIS performance in the first ten repeated LCs compared to verbal instructions, and completed the procedures with a lower complication rate.

In its current state, the iSurgeon is best used for performance assessment. The next step is to use the iSurgeon avatar to provide additional feedback, such as demonstrating how to move the wrist and elbow joints through real-time comparison with previously recorded expert-surgeon data. The iSurgeon provides the trainee with real-time feedback based on expert motion models, making it easier to perform the required task [20].

Such telementoring surgical tools with telestration and AR can be used in-room and remotely to proctor and assist with complex MIS procedures.

Finally, a study conducted by Kiani et al. in 2024 evaluated the educational value of a novel first-generation telestration tool used for surgical coaching [55]. The prototype utilizes an augmented reality platform and features four handheld functions: freehand annotation, cursor navigation, overlay, and manipulation of ghost (avatar) instrumentation and navigation on a remote monitor. The tool features a motion tracker and a Maryland grasper handle, creating a more realistic experience. The researchers evaluated the functional capability of the telestration device; most participants who tried it agreed that it can improve surgical coaching and would use it in an operating room. The development and testing of such telestration tools demonstrate how fast these technologies are advancing, particularly in enhancing surgical coaching with VR. The study provides a promising step forward in integrating the tool into real-time surgical environments.

#### Implementation of telestration with AR in emergencies

In remote areas or clinical environments lacking highly specialized surgical subspecialties, it is crucial to have access to a telementoring system that provides expert mentorship and surgical guidance during emergencies.

ARTEMIS (Augmented Reality Technology to Enable Remote Integrated Surgery) is an immersive AR-Virtual Reality telementoring infrastructure that enables experienced surgeons to remotely aid less experienced medical professionals in the field [51]. ARTEMIS provides immersive mixed reality visual aids by tracking a patient in real time and showing a reconstructed 3D point cloud in a VR environment. Expert surgeons can interact with the 3D point cloud representation of the patient, instruct the remote novice through real-time 3D annotations using hand maneuvers shown in AR through an avatar of the expert surgeon, and project small video clips of specific procedures in the AR space for the novice to follow [60]. It also enables expert mentors to annotate and draw pointers and markings visible to the trainee. The system evaluation was performed on mannequins and cadavers. It included six novices and five expert mentors. The mentors engaged in a total of 22 procedures across all sessions with novices, with two novices performing only one procedure (cricothyroidotomy) and the other four performing five back-to-back procedures each (cricothyroidotomy, dual-incision leg fasciotomy, femoral artery exposure, axillary artery exposure, and resuscitative thoracotomy). The research team observed all sessions, took notes, and analyzed procedure videos to uncover usability issues, usage patterns, communication, and coordination processes specific to immersive remote telementoring. Novices completed the procedures assigned to their sessions, including those they had never performed. The primary goal of the suggested telestration system with AR/VR was to ease the stress in emergency settings where there are no expert mentors to take over or monitor the novice handling of a critical situation. ARTEMIS allowed untrained medical personnel to perform complex surgeries on critical patients under direct guidance from remote experts [41].

#### Telestration with AR in overcoming spatial limitations in MIS and RAS

Traditionally, surgeons have had to be physically present at coaching sessions, which imposes considerable time and budget constraints. Telestration systems with AR have alleviated physical presence constraints without introducing more significant operative times or complication rates [28, 34, 35, 61, 62].

The System for Telementoring with Augmented Reality (STAR) was developed to address the decline in the surgical workforce, partially due to limited training opportunities. STAR is described as a novel platform that leverages an AR head-mounted display (ARHMD) worn by the trainee surgeon to display mentor-authored operative instructions. The trainee can visualize these expert instructions as 3D overlays directly onto their field of view of the patient’s body. To analyze STAR’s potential, expert surgeons mentored medical personnel remotely through a leg fasciotomy training procedure on cadaveric specimens. The experiment was conducted at two separate facilities. Twenty participants performed a leg fasciotomy training session on cadaveric specimens under one of two conditions: receiving remote instruction using the ARHMD system or receiving no external guidance beyond the initial consultation of the Advanced Surgical Skills for Exposure in Trauma course manual. A top-down camera captures the live video feed from the operative field at the trainee site and then sends it to the remote mentor. The remote mentor uses touch-based interactions to create technical annotations over this video feed. These annotations are returned to the ARHMD and appear as 3D imagery superimposed onto the trainee’s view of the operative field. These annotations can provide the local trainee with direct instructions from the remote mentor for the next surgical step.

The experiment tested the procedural and confidence outcomes between the two participant groups. Participants using STAR were reported to be more confident performing the procedure and to commit fewer errors than those without the mentoring tool [39].

Another telestration tool with AR, called VIPAR (Virtual Interactive Presence and Augmented Reality), was used to overcome spatial limitations and evaluate both subjective and objective system performance during an endoscopic third ventriculostomy with choroid plexus coagulation [31]. VIPAR is an iPad-based tool that allows surgeons to provide long-distance, virtual assistance wherever a wireless internet connection is available. Local and remote surgeons view a composite image of the video feeds at each station, enabling real-time intraoperative telecollaboration. Local and remote stations were established in Ho Chi Minh City, Vietnam, and Birmingham, Alabama, US, as part of an ongoing neurosurgical collaboration. Fifteen endoscopic third ventriculostomies with choroid plexus coagulation procedures have been performed using VIPAR between Vietnam and the US, with no significant complications observed.

#### Overcoming challenges of surgical training in the age of the COVID-19 pandemic

During the COVID-19 pandemic, access to the OR was limited. Therefore, there is an inevitable need for alternative surgical teaching methods that do not depend on traditional on-site teaching and proctoring. Future collaboration between surgeons of different expertise is also affected by travel restrictions and the inability to exchange knowledge and experience in surgical skills.

The developers of a cloud-based AR telesurgery platform, PROXIMIE, have addressed this limitation. PROXIMIE is already used in international surgeon-surgeon collaboration and training [37]. AR in PROXIMIE supplements a real-world environment with computer-generated sensory input, allowing users to interact with the environment. PROXIMIE has first been tested among undergraduate medical students. All students had access to the secure PROXIMIE streams on their computers, tablets, or phones. PROXIMIE camera rigs have been installed in general, vascular, plastic, and orthopedic ORs at the Royal Free Hospital, London, UK. A further camera was installed in the emergency theater. Students were invited to watch cases via e-mail, allowing them to view the operation remotely with in-built chat, audio, and webcam functions. They could participate virtually and learn about surgical procedures using affordable resources such as smartphones or computers.

PROXIMIE was not just designed for teaching undergraduate students. It allows multiple surgeons in remote locations to interact virtually, mimicing what they would experience collaborating in the same OR. This means they can physically show each other where to make an incision in real time or use physical gestures to illustrate a technique.

The PROXIMIE platform was used to treat two SARS-CoV-2-infected patients with Fournier’s gangrene in Jaber Al-Ahmad Hospital in Kuwait. The platform enabled the remote surgeons to guide the operating surgeon during extensive surgical debridement using voice, telestration, and hand gestures. The telestration technology with AR provided expert advice during these challenging cases without exposing additional team members to potential infection risk [40]. This approach provided the necessary treatment, ensuring high-quality procedures while minimizing the surgical staff’s exposure to risk.

In 2022, Youssef et al. described their preliminary experience with PROXIMIE and AR robot-assisted radical prostatectomy (AR-RARP). They concluded that PROXIMIE appears to be safe and effective for this approach, and the accuracy of 3D reconstruction is also promising [46].

#### Fifth generation (5G) wireless network and data transmission

Data transmission methods have suffered significant limitations over long distances, resulting in suboptimal data quality and quantity, as well as excessively long latency. However, early reports using the fifth-generation (5G) wireless network have been encouraging and are expected to further reduce latency.

AIS TeleSurgeon by the AIS Channel is a ‘plug-and-play’ device that does not require additional equipment other than a computer or tablet [38]. It processes and sends images, eliminating any added latency. With the help of 5G, it enables more stable data transmission, up to 100 times faster than its predecessors (10 GB/s), reduces latency to 1–2 ms, and increases simultaneous device connectivity by 100. Transmission via optical fiber is a viable option for short distances, particularly where established infrastructure is available. Current 5G technology has limitations, including specific hardware and structural costs, as well as safety concerns. However, the main advantage of 5G is the possibility of telementoring over exceptionally long distances with readily accessible hardware.

AIS TeleSurgeon was utilized in two patients requiring laparoscopic colorectal surgery. The mentor (Barcelona, Spain) communicated with the operating surgical team (Shanghai, China) and provided suggestions by drawing lines marking the surgical field area. There were no potentially unsafe situations or intraoperative complications during the procedures. The 5G technology enabled safe and efficient complex surgical procedures, utilizing telementoring with telestration in real time, with a remarkably high degree of surgical team satisfaction. It offers the possibility of working at exceedingly high transmission speeds, with a stable signal, and, above all, of practically eliminating the latency that the human brain can perceive [38].

## Discussion

Telestration systems with AR are working toward making safe surgery and efficient surgical training accessible to everyone, regardless of geographical distance. However, standardized surgical approaches and patient management protocols must be regulated to implement such educational tools. Today’s technology enables the rapid exchange of data and ideas, and surgical education and training must adapt accordingly. Nonetheless, the availability of novel approaches and data exchange has its risks. Therefore, it is essential to have evidence-based guidance in surgical practice and training while implementing modern technological advances [63].

The COVID-19 pandemic, with its lockdowns and travel restrictions, has demonstrated that the approach to surgical training and the performance of complex surgeries worldwide must be reassessed to ensure accessibility, regardless of the surgical experts’ distance or physical presence [4]. Telestration with AR provides a viable solution that eliminates the need for expert mentors to be on-site during the performance or teaching of advanced surgical procedures [63]. However, embedding telestration with AR into the standard surgical environment depends on financial incentives, favorable legislation, and collaboration with cybersecurity experts to ensure safety and cost-effectiveness. The role of telestration with AR may help optimize patient outcomes by facilitating expert input intraoperatively and reducing limitations caused by the need for transportation. Such technological applications should be studied to evaluate their feasibility and applicability.

There is a clear clinical need for enhanced access to surgical care in low-income countries [64]. Several barriers hinder the implementation of telementoring with telestration and AR, with the most inhibitive being the cost. Internet access and quality are poor in low-income countries, posing an additional technical challenge to the matter. Developing a secure framework to ensure that patients’ personal information and health-related data are encrypted and handled with care is vital.

Telestration tools with AR also aim to deliver professional instructions from academic high-volume settings to community clinics, supporting surgical training, performing complex procedures, and ensuring patient safety. However, the technology supporting telestration with AR has its safety concerns. These can include connection failures between the proctoring site and the OR [64], signal delays due to insufficient bandwidth [65], and susceptibility to cyberattacks [66]. Sensitive patient information must be transmitted securely across the internet, particularly between different institutions and across international borders.

Financial support is another critical aspect essential for adopting telestration systems with AR in surgical settings. Considering the economic implications of developing telementoring and telestration approaches in surgery, both on institutional and individual levels, is crucial. The primary financial concerns relate to the required equipment and network connection fees, which could pose difficulties, especially in hospitals with limited budgets and in developing countries where such support is most needed.

### Limitations

The presented review has several limitations. Only studies written in English were included, which limits our understanding of this field to studies written in other languages. Our search was limited to specific electronic databases: PubMed, ACM, IEEE, and Google Scholar. These electronic databases have been identified as the most relevant not only for medical but also for non-medical (technology-based) publications for the targeted topics of this scoping review. Non-medical electronic databases, such as IEEE and ACM, were used for the search. However, many studies here did not include the keywords "telestration" or "annotation" in their manuscripts, despite meeting the inclusion criteria as described and defined. Therefore, this represented a limitation in our exhaustive search, as some studies may have been missed because they did not specify the telestration used with the AR tool.

Due to the inclusion criteria, it was impossible to conduct a systematic review. Therefore, the decision was made to perform a scoping review. As a result, some selected studies may be subject to selection bias. However, specific inclusion criteria focusing on telestration with AR allowed a comprehensive review of the most relevant and recent studies. The included studies were heterogeneous in terms of study design, surgical procedures, skill levels, and communication systems. The development of new and advanced telestration and AR systems could account for the heterogeneity of the studies presented.

This scoping review identifies the promising potential of telestration with AR in surgical mentoring and training. However, the degree to which these benefits extend cannot be stated with certainty. Future studies must analyze the effects of telestration with AR in standardized and guided surgical and educational settings.

## Conclusion

Telestration with AR provides an advanced, geographically independent teaching tool by displaying instructions visually on the operative screen, sometimes obviating the need for on-site mentoring. Further research is needed to achieve the desired efficiency and proficiency of the assisting telestration tool with AR. Making the telestration tools with AR accessible is the next step in developing a global standard for surgical practice and training.

## Supplementary Information

Below is the link to the electronic supplementary material.

Supplementary file1 (DOCX 18 KB)

Supplementary file2 (DOCX 30 KB)

Supplementary file3 (DOCX 20 KB)

Supplementary file4 (DOCX 37 KB)
