# Supplementary material for: Telestration with augmented reality in minimally invasive and robotic-assisted surgery: a scoping review
Source: Surg Endosc. 2025 Nov 18;39(12):8000–13. doi: 10.1007/s00464-025-12380-2 (PMC12708694; doi:10.1007/s00464-025-12380-2)
Supplement: Supplementary file 1 — Supplementary file1 (DOCX 18 KB) [file 464_2025_12380_MOESM1_ESM.docx]

**Appendix 1:** Scoping Review Methodology

| Arksey & O´Malley | Levac et al. |
| --- | --- |
| Stage 1: Identifying the Research Question | Linking the purpose and research question |
| Stage 2: Identifying Relevant Studies | Balancing feasibility with breadth and comprehensiveness of the scoping process |
| Stage 3: Study Selection | Iterative approach (more than 1 reviewer) in selecting studies and extracting data |
| Stage 4: Charting the Data | Data charting form (numerical summary and qualitative thematic analysis) |
| Stage 5: Collating, Summarizing and Reporting the Results | Three parts: analysis, reporting and implications for future research and practice |
| Stage 6: Consultation Exercise as an option | Consultation as a necessary part of scoping review |

The Framework for Scoping reviews used in the current study according to Arksey & O'Malley and Levac et al. (15) (16).
