# Supplementary material for: Telestration with augmented reality in minimally invasive and robotic-assisted surgery: a scoping review
Source: Surg Endosc. 2025 Nov 18;39(12):8000–13. doi: 10.1007/s00464-025-12380-2 (PMC12708694; doi:10.1007/s00464-025-12380-2)
Supplement: Supplementary file 3 — Supplementary file3 (DOCX 20 KB) [file 464_2025_12380_MOESM3_ESM.docx]

**Appendix 3:** Search strategy for electronic databases

Limited to:

- English language

- Published from 2005 to February 2025

- Abstract available

**PubMed**

| **Search Number** | **Query** | **Search Details** |
| --- | --- | --- |
| **1** | (telestration[Title/Abstract]) | "telestration"[Title/Abstract] |
| **2** | (((video annotation[Title/Abstract]) OR (video annotation[Text Word])) OR (annotation[Title/Abstract])) OR (annotation[Text Word]) | "video annotation"[Title/Abstract] OR "video annotation"[Text Word] OR "annotation"[Title/Abstract] OR "annotation"[Text Word] |
| **3** | (telementoring[Title/Abstract]) OR (telementoring[Text Word]) | "telementoring"[Title/Abstract] OR "telementoring"[Text Word] |
| **4** | (augmented reality[Title/Abstract]) OR (augmented reality[Text Word]) | "augmented reality"[Title/Abstract] OR "augmented reality"[Text Word] |
| **5** | (minimally invasive surgical procedures[MeSH Terms]) OR (laparoscopy[MeSH Terms]) | "minimally invasive surgical procedures"[MeSH Terms] OR "laparoscopy"[MeSH Terms] |
| **6** | (robotic surgical procedures[MeSH Terms]) OR (Robot-Assisted Surgery [MeSH Terms]) | "robotic surgical procedures"[MeSH Terms] OR "Robot-Assisted Surgery"[MeSH Terms] |
| **7** | (surgical simulation [Title/Abstract]) OR (surgical simulation[Text Word]) | "surgical simulation"[Title/Abstract] OR "surgical simulation"[Text Word] |
| **8** | #1 OR #2 | "telestration"[Title/Abstract] OR "video annotation"[Title/Abstract] OR "video annotation"[Text Word] OR "annotation"[Title/Abstract] OR "annotation"[Text Word] |
| **9** | #3 OR #8 | "telementoring"[Title/Abstract] OR "telementoring"[Text Word] OR "telestration"[Title/Abstract] OR "video annotation"[Title/Abstract] OR "video annotation"[Text Word] OR "annotation"[Title/Abstract] OR "annotation"[Text Word] |
| **10** | #9 AND #4 | ("telementoring"[Title/Abstract] OR "telementoring"[Text Word] OR ("telestration"[Title/Abstract] OR ("video annotation"[Title/Abstract] OR "video annotation"[Text Word] OR "annotation"[Title/Abstract] OR "annotation"[Text Word]))) AND ("augmented reality"[Title/Abstract] OR "augmented reality"[Text Word]) |
| **11** | #5 OR #6 | ("minimally invasive surgical procedures"[MeSH Terms] OR "laparoscopy"[MeSH Terms]) OR  robotic surgical procedures"[MeSH Terms] OR "Robot-Assisted Surgery"[MeSH Terms] |
| **12** | #11 OR #7 | ("minimally invasive surgical procedures"[MeSH Terms] OR "laparoscopy"[MeSH Terms]) OR  robotic surgical procedures"[MeSH Terms] OR "Robot-Assisted Surgery"[MeSH Terms] OR "surgical simulation"[Title/Abstract] OR "surgical simulation"[Text Word])) |
| **13** | #12 AND #10 | ("minimally invasive surgical procedures"[MeSH Terms] OR "laparoscopy"[MeSH Terms]) OR  robotic surgical procedures"[MeSH Terms] OR "Robot-Assisted Surgery"[MeSH Terms] OR  "surgical simulation"[Title/Abstract] OR "surgical simulation"[Text Word] AND  (("telementoring"[Title/Abstract] OR "telementoring"[Text Word] OR ("telestration"[Title/Abstract] OR ("video annotation"[Title/Abstract] OR "video annotation"[Text Word] OR "annotation"[Title/Abstract] OR "annotation"[Text Word]))) AND ("augmented reality"[Title/Abstract] OR "augmented reality"[Text Word])) |

**Association of Computing Machinery (ACM)**

#1. [[All: telestration] OR [All: video annotation]]

#2. OR [All: or telementoring] AND [All: and augmented reality]

#3. AND [[All: and minimally invasive surgery] OR [All: laparoscopy]]

#4. AND [[All: robotic surgery] OR [All: robot-assisted surgery]]

#5. OR [All: and surgical training]

#6. [E-Publication Date: (01/01/2005 TO 10/31/2023)]

**Institute of Electrical and Electronics Engineers (IEEE)**

#1. ("All Metadata":telestration)

#2. OR ("All Metadata":video annotation)

#3. OR ("All Metadata":telementoring)

#4. AND ("All Metadata":augmented reality)

#5. AND ("All Metadata":minimally invasive surgery)

#6. OR ("All Metadata":laparoscopy)

#7. AND ("All Metadata":robot-assisted surgery) OR ("All Metadata":robotic surgery)

#8. OR ("All Metadata":surgical training)

**Google Scholar**

#1. telestration

#2. OR video annotation

#3. OR telementoring

#5. AND augmented reality

#6. AND minimally invasive surgery

#7. OR laparoscopy

#8. OR robotic assisted surgery

#9. OR robotic surgery

#10. OR surgical training
