# Supplementary material for: Telestration with augmented reality in minimally invasive and robotic-assisted surgery: a scoping review
Source: Surg Endosc. 2025 Nov 18;39(12):8000–13. doi: 10.1007/s00464-025-12380-2 (PMC12708694; doi:10.1007/s00464-025-12380-2)
Supplement: Supplementary file 4 — Supplementary file4 (DOCX 37 KB) [file 464_2025_12380_MOESM4_ESM.docx]

**Appendix 4:** Description of the included studies

|  | **Baseline Characteristics of the Included Studies** | | | | | | | |  |
| --- | --- | --- | --- | --- | --- | --- | --- | --- | --- |
| **Reference** | **Year** | **Country** | **Intervention** | **Type of telestration tool** | **Distance** | **Factors/Outcomes** | **Price** | **Participants** | |
| Bruschi et al. | 2005 | Italy | Assessment of the telementoring approach in laparoscopic adrenalectomy in a single center. | The remote operating site had a high-resolution video monitor with speakers, a multidirectional microphone, and a Telestrator sketchpad (eZmeeting, Sigma Designs, Inc., Milpitas, CA). | 430 km | Technical difficulties, time delay of the communication, surgery time, mean blood loss, blood transfusion, postoperative complications, length of stray. | Not reported | 1 mentor, 1 trainee, 8 surgeries | |
| Agarwal et al. | 2007 | USA | Implementation of the RoboConsultant telementoring system during laparoscopic and endoscopic urologic procedures. | Remote presence robotic (RP-7) telementoring system with audial and visual feedback, portable laptop control station, and wireless connectivity allowing the consultant to be mobile, interacting with the operating room team remotely from any location with broadband connectivity. | 8 km | RoboConsultant functionality including navigation, zoom capability, examination of external and internal endoscopic camera views, and telestration. | 150 000$ | 1 senior, 1 junior surgeon, 5 procedures | |
| Ali et al. | 2008 | USA | Developing of a video algorithm for 3-D telestration in the Da Vinci visual field. | Functional prototype of a 3-D telestration system that integrates into the imagining system of the Da Vinci surgical robot. | In-room and remotely (mentor station) | The prototype of 3-D telestration was reported to have a clear image and function correctly in the Da Vinci screen display. The time required for the task and accuracy were measured using only inanimate models. | Not reported | 3 trainees, 1 mentor, 33 task-repetitions per trainee | |
| Schlachta et al. | 2009 | Canada | Comparison of three phases of mentoring: scrubbing in and direct mentoring, verbal mentoring without scrubbing in, and remote telementoring in elective laparoscopic colon surgeries. | Remote telementoring system with high-resolution touch-sensitive annotator screen on mentor's size. | 60 km | Conversion rate, surgery time, length of stay, median number of lymph nodes resected, and technical difficulties of the remote mentoring system. | Not reported | 1 mentor surgeon, 2 trainees, 20 surgeries | |
| Hinata et al. | 2014 | Japan | 3D telementoring system with telestration and AR for da Vinci S surgical system in radical prostatectomy compared to direct mentoring. | Novel 3D high-definition telementoring system with an overview of the operating room, annotation function, and 2-channel audio feed with bidirectional connectivity. | 230 km | Reliability of the telementoring system (errors of communication links, latency, failure of the network), patient outcomes (operating time, estimated blood loss, blood transfusions, complication rate, positive surgical margin, continence rate at three months after surgery), learning curve. | Not reported | 4 trainees, 1 mentor, 120 performed surgeries | |
| Ponsky et al. | 2014 | USA | Two remote telementoring systems to provide two-way communication between mentors and mentees while performing video-assisted thoracoscopic lower lobectomy, gastric stimulator placement, and laparoscopic inguinal hernia repair in pediatric patients were compared. | The first telementoring system consisted of store-bought equipment that connected the operating room laparoscope to a Skype™ (Microsoft, Redmond, WA) connection (used in 1 case) without a telestration feature. The second was a proprietary telementoring robot, Karl Storz Endoscopy-America, Inc. VisitOR1(®) (Karl Storz GmbH & Co. KG, Tuttlingen, Germany) (used in 5 cases) with telestration feature. | 2.140 km | The first telementoring system with Skype did not have a telestration option and was not adequately secure. The VisitOR1 telementoring robot enabled high-resolution video communication, had telestrator capacity, and allowed pointing during the procedure. | Not reported | Not specified | |
| Forgione et al. | 2015 | Italy | Establishing a new remote learning method with a telestration tool with AR called OR1 Smartconnect to provide safe teaching for laparoscopic colorectal surgery. | OR1 Smartconnect is a telestration mentoring tool with AR that provides real-time video streaming of the laparoscopic camera view, an audio communication link between the mentor and the operating team, and a video view of the operating theater. The operative image was displayed on a touch-sensitive annotator screen on the mentor's desk with enabled telestration features. | 2.868 km | The success of the surgical procedures and follow-up of the next 25 procedures performed by the same surgeon. | Not reported | 1 trainee, two procedures | |
| Shin et al. | 2015 | USA | Application of Connect software for remote mentoring with telestration and AR in da Vinci Si robotic surgery. | Using a mouse or trackpad, the Connect software enables the mentor to draw on his screen. The line drawings are then viewable to the trainee as an overlay onto the surgical field in the surgeon's console. | In-room and remotely 350 m | Rating of Connect software in anatomical recognition, improving surgical skills, surgical confidence, safety, ease of use, and telestration helpfulness.  Technical aspects like latency, Connection setup time, dropped sessions, and sessions with data loss in remote cases between wired and wireless connections. | Not reported | 11 trainees, 10 mentors | |
| Davis et al. | 2016 | USA | System performance evaluation of Virtual interactive presence and augmented reality (VIPAR) in a remote setting while performing endoscopic third ventriculostomy with choroid plexus coagulation. | VIPAR is an iPad-based telementoring tool that views a composite image of video feeds at remote and local stations, allowing visual demonstration and telecollaboration from both sides (mentor and trainee). | 14.887,67 km | Audio and video composite latency, accuracy analysis, and clinical utility analysis via questionaries. | $14.930,39 for 1 calendar year | 15 surgeries, 1 mentor, 1 trainee | |
| Jarc et al. | 2016 | USA | Comparison of 2D telestration with three 3D novel proctoring tools (3D pointer, 3D cartoons hands, 3D instruments) in a dry lab setting. | 3D semitransparent tools with the ability to draw line annotations on the stereoscopic endoscopic images streamed from the system's video outputs. | In-room; can be used remotely | Assessing 3D proctoring tools in improving surgical/technical skills, helping delineate anatomic structures, improving confidence, allowing safe completion of tasks, working smoothly, being easy to use, being helpful, and being more helpful than 2D telestration. | Not reported | 26 proctor-trainee pairs | |
| Snydermann et al. | 2016 | USA | Implementation of a telementoring system with telestration and AR in endoscopic endonasal skull base surgeries | In-Touch VisitOR_1_ (Karl Storz; Tuttlingen, Germany) robot with additional visualization characteristics (remote zoom, panoramic views in addition to the S-video feeds), laser pointer, and telestration feature. | 7.260 km In-room could be used remotely | Audio, video, and telestration quality assessment of the implanted telestration tool with AR. | Not reported | 10 cases | |
| Kowalewski et al. | 2017 | Germany | Implementation of iSurgeon in assessing the accuracy of the novel training software with AR in laparoscopy and developing a virtual surgical avatar for visual feedback with AR. | Sensor- and expert-model-based laparoscopic training system with AR and telestration options called iSurgeon. | In-room; can be used remotely | Validity and reliability of iSurgeon through comparison of iSurgeon parameters (time, path length, average speed, angular path, number of movements, and number of movements per second) between three groups and comparing iSurgeon parameters with OSATS scores. | Not reported | 210 novices, 10 intermediate-level surgeons, 10 experts | |
| Luck et al. | 2017 | UK | The aim was to assess whether AR enhances undergraduate surgical education. PROXIMIE camera rigs have been installed in general, vascular, plastic, and orthopedic theatres. A further camera was installed in the emergency theatre. Students were invited to watch cases via email, allowing them to view the operation remotely with in-built chat, audio, and webcam functions. They could participate virtually and learn about surgical procedures using affordable resources such as smartphones or computers. | A cloud-based AR telesurgery platform that supplements a real-world environment with computer-generated sensory input, allowing users to interact with the environment. | Remotely, not specified | Pre- and post-pilot study questionaries about the PROXIMIE AR platform and its use. | Not reported | 114 students, 26 consulting surgeons, 23 surgeries | |
| Jarc et al. | 2017 | USA | Use the 3D ghost tools to mentor trainees during training on the da Vinci Xi™ Surgical System on a live porcine model. | A 3D semi-transparent tool with the ability to draw line annotations on the stereoscopic endoscopic images streamed from the system's video outputs. | In-room; can be used remotely | Exploitation of tool's features through assessment of hand movements and button presses.  3D movements (3DMOVE), wristedness (WRIST), trigger use (TRIG), and bimanual instruction (BIMAN). | Not reported | 7 trainees and 6 proctors | |
| Lacy et al. | 2019 | Spain | Testing of AR telementoring tool called AIS TeleSurgeon remotely in two laparoscopic colorectal surgeries. During the procedures, the surgeons and the mentor communicated in real-time through an audiovisual system providing the same internal and operating field images. The mentor could also draw on the screen of a laptop, which the surgeons could see on their screens (telestration). | AIS TeleSurgeon is a 'plug and play' AR device that processes and sends images, removing any added latency. With the help of 5G, it enables more stable data transmission and up to 100 times faster than its predecessors. | **4.911,97 km** | Postoperative complications and technical aspects of the telestration tool like latency, stability of the signal, image quality, and transmission speed. | Not reported | 2 laparoscopic colorectal surgeries, number of participants not specified | |
| Rojas-Muñoz et al. | 2020 | USA | Comparison of the effects of telementoring with System for Telementoring with Augmented Reality (STAR) with the effects of no external guidance other than initial consultation of the Advanced Surgical Skills for Exposure in the Trauma course manual in 20 trainees performing leg fasciotomy on cadavers. | The System for Telementoring with Augmented Reality (STAR) is a novel platform that leverages an AR head-mounted display (ARHMD) worn by the trainee surgeon to display mentor-authored operative instructions. | Remotely, not specified | Number of errors, procedure completion time, and self-reported confidence scores. | Not reported | 20 trainees, number of expert mentor surgeons not specified | |
| Alyaqout et al. | 2021 | Kuwait | Implementation of PROXIMIE AR platform in 2 complex cases of Fournier's gangrene in COVID patients due to the complexity of cases and necessity of interdisciplinary surgical treatment. | The PROXIMIE platform enabled remote surgeons to guide the operating surgeon during extensive surgical debridement using voice, telestration, and hand gestures. | Remotely between two surgical teams, not specified | Successful use of the PROXIMIE in complex cases requiring interdisciplinary surgical treatment and consultation. | Not reported | 2 surgeons | |
| Gasques et al. | 2021 | USA | Assessment of the use of ARTEMIS (Augmented Reality Technology to Enable reMote Integrated Surgery), a mixed-reality system in remote telementoring in emergencies such as cricothyroidotomy, dual-incision leg fasciotomy, femoral artery exposure, axillary artery exposure,  and resuscitative thoracotomy performed on cadavers and mannequins. | ARTEMIS: an immersive AR-Virtual Reality telementoring system that allows experienced surgeons to aid less experienced medical professionals remotely. It provides Mixed Reality immersive visual aids by tracking a patient in real-time and showing a reconstructed 3D point cloud in a VR environment. | Remotely, not specified. | Technical issues, feedback from experts and novices regarding the difficulty of usage and perception, as well as benefits of the telementoring system. | Not reported | 5 mentors, 6 novices, 22 procedures | |
| Wild et al. | 2022 | Germany | Application of the iSurgeon system in laparoscopic cholecystectomy in the box trainer. | The iSurgeon telestration system of AR-based video assistance allows the combination of verbal instructions during training with visual instructions on the laparoscopic monitor. | In-room; can be used remotely | GOALS, OSATS scores, and subjective NASTA-TLX questionaries. | Not reported | 60 students were randomized in two groups (with and without iSurgeon) | |
| Yang et al. | 2022 | USA | Evaluating Cardiovascular Surgical Planning in Mobile Augmented Reality. | Mobile AR cardiovascular surgical planning through AR with tools like virtual annotation and slicing of models. | In-room | Usability and usefulness of the tool. | Not reported | 6 medical experts | |
| Neves López et al. | 2022 | Portugal | Telementoring and telestration with AR for a basic surgical skill course. | Smart glasses mounted with a headband with AR. The students perform different suture exercises while a mentor provides remote feedback. A 2-D mouse was used for visual instructions, such as pointing and adding lines, text, and objects to the student's screen. The audio instructions were provided through FaceTime. | In-room; can be used remotely | OSATS, learning experience, confidence, self-evaluation. | Not reported | 20 students. Number of mentors is not specified | |
| Müller et al. | 2022 | Germany | Robust Hand tracking for surgical telestration and augmented Reality visualization of surgical hands. | This VR telestration approach relies on a camera that continuously captures the hand of the mentor who observes the procedure on-site or remotely. The real-time hand segmentation is shown with high accuracy on the screen. | In-room; can be used remotely | Hand detection accuracy, assessment of speed, real-time hand localization. | Not reported | 3 mentors and 705 videoframes | |
| Youssef et al. | 2022 | United Kingdom | Augmented reality robot-assisted radical prostatectomy with PROXIMIE: Preliminary clinical experience. | The PROXIMIE telestration tool was used under the surgical context of a radical prostatectomy. The preliminary clinical experience shows that this system seems to be safe and effective, having an accurate 3-D reconstruction. | Remotely, not specified | Parameters assessed included efficiency: video quality, audio quality, and ease of use.  Safety, teaching, and prospects. | Not reported | 2 senior urology fellows were mentees to 2 expert urology mentors | |
| Maria et al. | 2023 | France | Supporting collaborative discussions in Surgical teleconsulting through augmented reality head-mounted displays. | AR Head Mounted Display (AR-HMD) with telestration and annotation tools. This telecollaboration system allows surgeons to consult with radiologists when struggling to find a non-visible structure during a surgery previously identified in a preoperative image.  The radiology experts can provide life annotations in the radiological images, which the surgeon can visualize through the AR-MHD while performing the surgery. | In-room; can be used remotely | Evaluation of different settings of the tool with surgery and abdominopelvic surgery scenarios:  -Fixed display + single user  -Mobile display + single user  -Mobile display + dual user  Parameters assessed included task completion time, communication balance, communication through deictic referencing, use of a secondary display, and perceived usefulness. | Not reported | 12 experts in 6 different scenarios | |
| Felinska  et al. | 2023 | Germany | The effect of telestration using the iSurgeon system with AR on gaze behavior during MIS training. | iSurgeon telestration system with AR and Pupil Core eye tracking glasses to capture instructor and trainee gaze behavior. | In-room; can be used remotely | The time needed to complete the task, number of errors, OSATS, NASA-TLX, gaze latency, gaze convergence, and collaborative gaze convergence. | Not reported | 40 students were randomized into two groups | |
| Rodrigues et al. | 2023 | USA and Canada | Comparison of video telestration and written feedback vs. feedback alone of ERCP technical skills. | Video Telestration consisted of a video narration with superimposed visual cues to show the ideal technique. | In-room | Reported utility, perceived helpfulness. | Not reported | 24 trainees, number of mentors not specified | |
| Cizmic et al. | 2023 | Germany | Implementation of the iSurgeon telestration device for ex vivo laparoscopic cholecystectomies. | The iSurgeon telestration system with AR allows to provide real time screen guidance in addition to verbal instructions. This system was used in the context of surgical simulation of laparoscopic cholecystectomies. | In-room; can be used remotely | GOALS, OSATS, Critical View of Safety, complication rate. | Not reported | 40 students were randomized into two groups | |
| Huettl et al. | 2023 | Germany | HoloPointer telestration system influences surgical performance, specifically on laparoscopic cholecystectomies. | HoloPointer is a telestration tool that allows real-time annotations on a laparoscopic monitor for intraoperative guidance.  This system operates with verbal commands and head movements. | In-room; can be used remotely | GOALS, CVS, operation time, quality of assistance, user-friendliness. | Not reported | 15 trainees, 13 trainers, 29 surgical teams, and 32 procedures | |
| Shafa et al. | 2023 | Canada | Use of a multimodal videoconference platform to coach surgeons and improve intraoperative performance. | Remote coaching with telestration was conducted using the Zoom platform. Annotations were made by free-hand drawing directly onto the surgical field. | Remotely | Intraoperative performance assessment tool (IPAT),  Evaluation survey for educational value, technical platform usability and feedback with a 5-point Likert scale. | Not reported | 2 couch surgeons and 6 mentees | |
| Takemasa et al. | 2023 | Japan | Clinical feasibility of tele-proctoring for minimally invasive surgery across Japan. | The tele-proctoring system used was TELEPRO, which also provided live telestration with annotation tools. The server recorded latency between two locations by detecting the use of the annotation pen. | Remotely | Total latency between two locations, questionnaires for quickness and quality of communication and usefulness in surgical education. | Not reported | Seven hospitals were connected via tele-proctoring to the Sapporo Medical University (1 expert surgeon) | |
| Liu et al. | 2024 | China | Exploration of the application of augmented reality technology for teaching spinal tumor´s anatomy and surgical techniques. | The use of virtual reality and augmented reality technology to present the surgical process of percutaneous vertebroplasty for spinal tumors. The reconstruction of surgical instruments, multiple person collaboration and position sharing was also possible. Use of 3Ds Max software and the HoloLens 2 HMD. | In-room | Survey for satisfaction, teaching quality, benefits of using the AR tools, understanding of the general steps of the surgery and anatomy. | Not reported | 2 groups of medical students: 12 vs 13 students | |
| Kiani et al. | 2024 | Canada | Evaluation of usability, ergonomics and educational value of a first-generation handheld wireless telestration platform. | Prototype of a first-generation telestration tool customized for a use in surgical couching to optimize feedback and training. Allows the tutors to interact with the surgical display field in intraoperative and postoperative settings. The four core functions are free hand annotation, cursor navigation, overlay and manipulation (rotation) of a ghost avatar (instrument) and hand-held video feed navigation on a remote monitor. | In-room, 7m between the tracker and the lighthouse | Usability and ergonomics were evaluated with a validated system usability scale and a 5-point Likert scale.  The perceived educational value of the device was also assessed with the 5-point Likert scale. | Not reported | 9 surgeons and 1 senior resident | |
| Tanaka et al. | 2024 | Tokyo | Intraoperative telestration system in endoscopic transsphenoidal surgery (ETS). | Telestration system used during opening of the sellar floor in ETS.  Two telestration systems were used: NUCLeUS and ADMENIC ANNOTATOR. The mentor made anatomical annotations in the tablet which were then transferred back to the telestration system. | In-room | Duration and accuracy of the tasks using a ETS model, concordance rate between the planned and actual window opening area between groups. | Not reported | 18 novice surgeons | |
